# New Species of *Oochoristica* Lühe, 1898 (Cestoda: Linstowiinae) from *Sceloporus ochoterenae* (Reptilia: Sauria: Phrynosomatidae) in Central Mexico: Approach to the Phylogenetic Relationships of the Genus with Molecular Evidence: *Oochoristica Maccoyi* N. Sp. (Cestoda: Linstowiidae) from *Anolis gingivinus* (Sauria: Polychrotidae) Collected in Anguilla, Lesser Antilles

**DOI:** 10.1007/s11686-025-01046-7

**Published:** 2025-05-16

**Authors:** Andrés Velázquez-Brito, Luis García-Prieto, Uriel Garduño-Montes de Oca, Virginia León-Règagnon

**Affiliations:** 1 Laboratorio de Helmintología, Departamento de Zoología, Instituto de Biología, Universidad Nacional Autónoma de México, Circuito Zona Deportiva s/n, Ciudad Universitaria, Copilco, Ciudad de México, Coyoacán, CP 04510 México; 2 Estación de Biología Chamela, Instituto de Biología, Universidad Nacional Autónoma de México, A. P. 21, San Patricio, Jalisco, CP 48980 México

**Keywords:** Morphology, Endemic lizard, Phylogeny, Cyclophyllidea, Taxonomy

## Abstract

**Purpose:**

To describe a new species of *Oochoristica* Lühe, 1898, an anoplocephalid cestode included in Linstowiinae (Cyclophyllidea), parasitizing *Sceloporus ochoterenae* Smith in Zoyatepec, Guerrero, Mexico.

**Methods:**

All specimens were fixed with 4% hot formalin. A morphologic study was made using stained material under light microscopy. Some specimens of a second species (collected in *Sceloporus grammicus* from La Malinche, Tlaxcala, fixed in 100% ethanol, were also stained and studied morphologically; others were subject of DNA sequencing and phylogenetic analysis.

**Results:**

A new species of this genus is described of the intestine of an endemic lizard *S. ochoterenae*. *Oochoristica guerreroensis* n. sp. shares a distinctive morphological feature (mature proglottids wider than long) with only 13 species of genus. However, *O. guerreroensis* n. sp. can be differentiated by: strobilar size (4.29–7.62 mm), number of testes (48–55), shape of vitellaria (ovoid) and number of ovarian sublobes (3–5), as well as by their geographical distribution (Neotropical region of Mexico), and type of host. Based on molecular evidence of nuclear 28*S rDNA* gene, we analyze for the first time the phylogenetic position of one species of *Oochoristica* (*O. scelopori*) within Anoplocephalidae as well as their relationships with other genera of Cyclophyllidea.

**Conclusions:**

The new species is the first described in association with a phrynosomatid lizard in the Neotropical realm, and the ninth recorded in Mexico. Molecular data obtained in our study seem to confirm the independence *Oochoristica* and *Mathevotaenia*.

## Introduction

*Oochoristica* Lühe, 1898, an anoplocephalid cestode genus included in Linstowiinae (Cyclophyllidea), comprises almost 100 parasitic species of reptiles and mammals worldwide [1, 2]. Thirty-four of these species have been recorded in the American continent: 16 from the Neotropical realm and 18 from the Nearctic, all associated with reptiles. Particularly, for Phrynosomatidae reptiles, four Nearctic species are known: *Oochoristica scelopori* Voge and Fox, 1950, *O. parvovaria* Steelman, 1939, *O. phrynosomatis* (Harwood, 1932) Loewen, 1940, *O. macallisteri* Bursey and Goldberg, 1996 [3, 4]. However, in the Neotropical region no *Oochoristica* species associated with this family of lizards have been described.

As a part of the project “The amphibians and reptiles and their parasites of Mexico, a megadiverse country”, carried out between 2001 and 2012, were collected 12 tapeworm specimens belonging to *Oochoristica*. Cestodes were found in intestine of “Ochoterena lizard” *Sceloporus ochoterenae* Smith in the State of Guerrero; the geographic distribution of this lizard includes the Mexican States of Morelos, Michoacán, Puebla, Oaxaca, and Guerrero [5, 6]. The aim of this work is two folds: (1) to describe morphologically the first species of this genus associated with a phrynosomatid lizard in the Neotropics, comparing it with all the species described in American continent, as well as with other species with similar characteristics distributed worldwide, and (2) to establish the phylogenetic position of the genus *Oochoristica* (based on an independent collection of *O. scelopori*) with the 28*S rDNA* nuclear gene within Cyclophyllidea in general, and Anoplocephalidae in particular.

## Materials and Methods

### Collection and Morphological Study of Oochoristica Species

During 2005, sixteen specimens of *S. ochoterenae* were collected by hand in Zoyatepec (17°19’43’’N; 99° 33’ 09’’W), Guerrero, Mexico, for helminthological study. A second species of lizard (three female gravid specimens of *Sceloporus grammicus* Wiegmann), were collected in 2022 at the foothills of La Malinche volcano, Tlaxcala, México (19° 14’ 41” N; 98° 00’ 08"W). Lizards were euthanized with an intraperitoneal overdose of sodium pentobarbital, and reviewed for helminths following Lamothe-Argumedo [7]; briefly, cestodes were removed from the intestine and washed in 0.65% saline. Tapeworms from Zoyatepec were fixed with 4% hot formalin, while the samples of La Malinche in absolute ethanol (genetic study) and 70% hot ethanol (morphological study). Specimens of both morphospecies were stained with Mayer’s paracarmine and Delafield’s hematoxylin, cleared in methyl salicylate, and mounted in permanent preparations with Canada balsam. Morphological analysis was performed under light microscope; measurements, obtained with an ocular micrometer adapted to microscope, are given in micrometers, unless otherwise noted; we present range and mean, standard deviation, and sample size (n). Line drawings of the tapeworms were made with the aid of a camera lucida adapted to a microscope, final editing was done in (Adobe Systems, San Jose, CA, USA) using a Wacom One drawing tablet. Prevalence of infection was calculated according to Bush et al. [8]. Holotype and paratype material were deposited in the Colección Nacional de Helmintos (CNHE), Instituto de Biología, Universidad Nacional Autónoma de México, Mexico City.

### Genetic Study of Oochoristica Scelopori

A part of tapeworm previously fixed in absolute ethanol were placed in 1.5 ml microcentrifuge tube. Tissue digestion and DNA extraction was achieved using EZ-10 Spin Column Genomic DNA Minipreps Kit, Bio Basic Inc. (Ontario, Canada), according to the manufacturer’s instructions. Large Ribosomal Subunit (28*S*) rDNA gene was amplified with primers forward primer 391 (5 -AGCGGAGGAAAAGAAACTAA) and reverse primer 501 (5 -TCGGAAGGAACCAGCTACTA) [9]. Polymerase Chain Reaction (PCR) was prepared in a total volume of 25 µL containing: 16.39 µl ddH20, 2.5 µl buffer, 2.5 µl MgCl2, 1 µl of each primer, 0.56 µl dNTPs, 0.05 µl Taq polymerase, and 1 µl total genomic DNA sample. Amplification profile started with an initial denaturing of 95° C for 5 min, then 40 cycles with a first step of 94° C for 30s, a second step of 55° C for 45 s and a third step of 72° C for 1 min, and a final extension of 72° C for 10 min. PCR reaction products were visualized by agarose gel electrophoresis. Then, sequencing was performed at the Laboratorio Nacional de la Biodiversidad (LANABIO), Instituto de Biología, UNAM, in an ABI PRISM 3730 sequencer (Applied Biosystems, Carlsbad, CA). Sequence obtained was assembled and edited in Geneious Pro ver 5.1.7 software. Blast GenBank was searched to taxonomically assign the generated cestode sequences.

### Phylogenetic Analysis

For the phylogenetic analysis we used sequences available in GenBank of species belonging to Cyclophyllidea; then, we constructed a matrix with a total of sixty-three sequences with 28*S rDNA* gene, for the multiple sequences alignment we used the online version of MAFFT v.7 [9] with default parameters and made a final manual editing of the endpoints in Mesquite v. 3.51 [11]. JModeltest v. 3.0 was used to infer the best evolution model [12]. Bayesian inference was performed with the program MrBayes v. 3.2.7 [13]. The settings were fixed as follows: 2 simultaneous runs with 4 Markov Chains Monte Carlo (MCMC) for 10 million generations, sampling every 1000 generations, a heating parameter value of 0.2, and a ‘‘burn-in’’ of 25%. The convergence statistics were checked using Tracer v. 1.7 [14]. A 50% majority-rule consensus tree representing the posterior probability distribution of clades was produced for the sampled trees. The tree was visualized and edited in Fig Tree v.1.4.4 [15].

## Results

**Anoplocephalidae** Cholodkovky, 1902.

***Oochoristica*** Lühe, 1898.

**Fig. 1:**
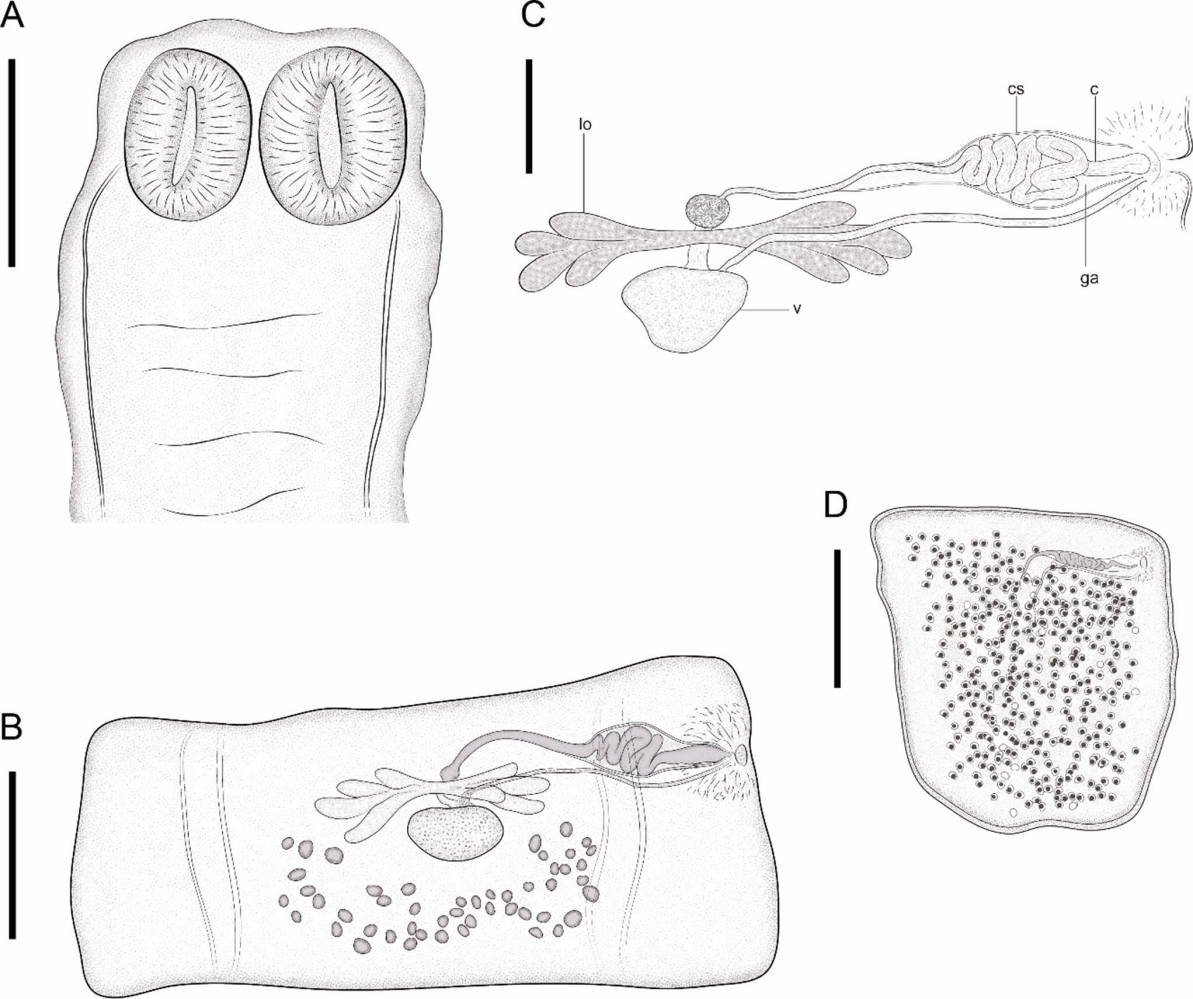
*Oochoristica guerreroensis* n. sp. Line drawings, adult tapeworm parasitizing *S. ochoterenae*. **A** Scolex and suckers, ventral view. **B** Mature proglottid with male-female reproductive system. **C** Cirrus (c), cirrus sac (cs), vitellarium (v), lobulate ovary (lo) and genital atrium (ga). **D** Gravid proglottid. Figures scales A, B and D is 150 μm and C is 60 μm

**Fig. 2:**
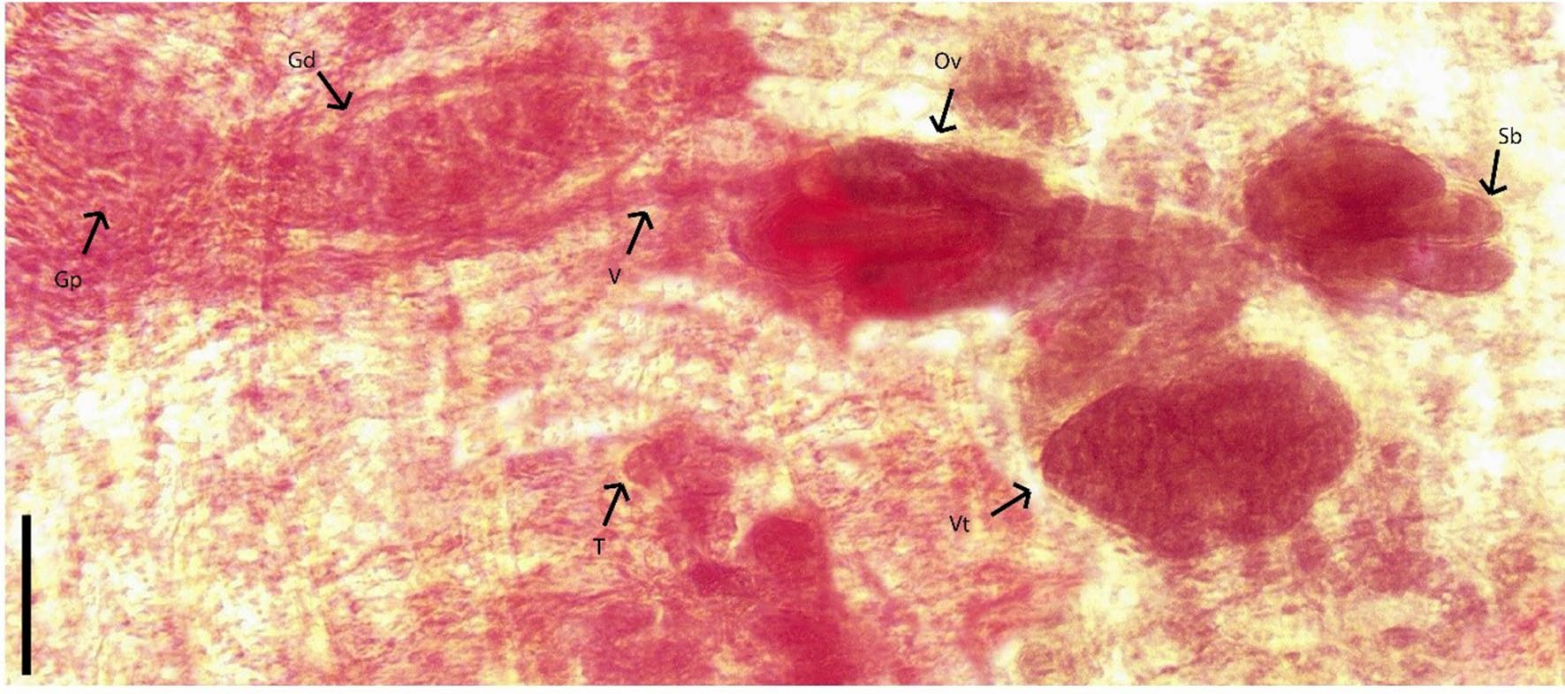
*Oochoristica guerreroensis* n. sp. Light micrograph: main structures indicated by arrows followed by abbreviations; genital pore (Gp), genital ducts (Gd) vagina (V), testes (T), bilobed ovary (Ov), conformed by sublobes (Sb) and ovoid-shaped vitellarium (Vt) located in central part of mature proglottid. Scale bar 40 μm

***Oochoristica guerreroensis* n. sp.** Figs. 1 and 2.

### Taxonomic Summary

#### Type Host

Ochoterena’s lizard, *Sceloporus ochoterenae* Smith, 1934.

#### Type Locality

Zoyatepec (17°19’43’’N; 99° 33’ 09’’W), Guerrero, Mexico.

#### Site of Infection

Intestine.

#### Prevalence of Infection

6%, (1/16 lizards).

#### Etymology

The new species name is given from the Mexican State where was collected, which has a great diversity of flora and fauna representative of Mexico.

#### Specimens Deposited

Holotype, Colección Nacional de Helmintos (CNHE 12223); paratypes (CNHE 12224).

*ZooBank registration*: http://zoobank.org/urn:lsid:zoobank.org:pub:72FA84EF-86BE-4E0B-8CB0-A9ADD97D5110.

**Description** based on twelve gravid tapeworms: Cyclophyllidea: Anoplocephalidae (Linstowiinae). Dorsoventrally flattened worms; strobila acraspedote, containing 31–39 (34.5 ± 2.276, *n* = 12) proglottids; 4.478–7.393 mm (5.473 ± 0.962, *n* = 12) total body length and 621–835 (719 ± 0.61, *n* = 12) maximum width at level of mature proglottids. Proglottization distinguishable from immature to gravid proglottids. Strobila contains 25–31 (27.333 ± 1.614, *n* = 12) immature, 3–4 (3.333 ± 0.492, *n* = 12) mature and 2–6 (3.916 ± 1.164, *n* = 12) gravid proglottids. Scolex unarmed and rounded; 271–473 (373 ± 0.53, *n* = 12) length by 318–546 (434 ± 0.65, *n* = 12) width, with four thick oval suckers, 201–301 (241 ± 0.27, *n* = 12) length and 127–227 (179 ± 0.26, *n* = 12) width (Fig. 1A). Neck extremely short 36–63 (50 ± 0.10, *n* = 12) in length. Mature proglottids wider than long, 627–755 (675 ± 0.37, *n* = 9) width and 182–354 (265 ± 0.49, *n* = 12) length (Fig. 1B). Gravid proglottids longer than wide; 546–910 (724 ± 0.105, *n* = 12), by 501–728 (619 ± 0.57, *n* = 12) respectively (Fig. 1D). Subspherical and oval testes, 48–55 per proglottid, 11–17 (13 ± 0.01, *n* = 12) × 17–23 (19 ± 0.01, *n* = 12); distributed in one cluster, posterior to vitelline gland, occupying the mid region of mature proglottids. Some testes reach ovarian lobes, but not cirrus sac. (Fig. 1B). Pre-equatorial genital pore, irregularly alternating (Fig. 1B). Small and generally thin cirrus. Cirrus sac 62–97 (80 ± 0.12, *n* = 12) long by 26–68 (41 ± 0.14, *n* = 12) wide, passing between osmoregulatory channels (Figs. 1B- C and 2). Deferent duct forming several loops within cirrus sac (Figs. 1C and 2). Bilobed ovary, 164–218 (196 ± 0.17, *n* = 12) length by 28–81 (45 ± 0.14, *n* = 12) width, located in the proglottid central zone; left lobe with 4 – 5 sub–lobes (4.58 ± 0.51, *n* = 12) and right lobe with 4–6 (4.333 ± 0.65, *n* = 12) (Figs. 1B-C and 2). Seminal receptacle small, thin and tenuous. Vitellaria central, ovoid, post-ovarian, 34–67 (48 ± 0.10, *n* = 12) length by 65 ± 91 (22 ± 0.32, *n* = 12) width. Vagina passing over ovarian lobes, running parallel to cirrus sac and open into genital atrium posterior to cirrus; vaginal sphincter absent. Uterus ephemeral. Numerous ovigerous capsules containing one egg, distributed throughout gravid proglottid; each egg 10–16 (12 ± 0.20, *n* = 12) length by 13–21 (16 ± 0.20, *n* = 12) width.

***Remarks*** Tapeworms collected in *S. ochoterenae* were assigned to *Oochoristica* according to the diagnostic characteristics of the genus presented by Beveridge [16].

*Oochoristica guerreroensis* n. sp. is characterized by having mature proglottids wider than long; this trait are the main difference among the new species and 87 species of the 100 currently included in the genus [2, 3]. The new species shares this feature only with 13 species around the world. Specifically, for the American continent, only five species present this morphology, three distributed in the Neotropical realm: *Oochoristica freitasi* Rego and Ibañez, 1965; *O. maccoyi* Bursey and Goldberg, 1996 and *O. leonregagnonae* Arizmendi-Espinosa, García-Prieto and Guillén-Hernández, 2005, and two in the Nearctic: *O. bezyi* Bursey and Goldberg, 1992 and *O. islandensis* Bursey and Goldberg, 1992 [17].

One of the species of the Neotropical realm (*O. freitasi*), closely resembles to *O. guerreroensis*; however, total body length of the new species is smaller (4.4–7.3 mm vs. 40 mm), and also differs in the number of proglottids, since *O. guerreroensis* n. sp. has 31–39 meanwhile *O. freitasi* has a total of 60. In addition, absence of neck in *O. freitasi* allows distinguish it from the new species, which has a relatively short neck (0.27–0.55) as well as an ovoid and not round vitellarium showed by *O. freitasi*. Finally, *O. freitasi* was described in Peru with *Dicrodon heterolepis* (Teiidae) as the type host [18]. On the other hand, *O. maccoyi* has a smaller number of testes compared to *O. guerreroensis* n. sp. (10–16 vs. 48–55, respectively). Furthermore, total body length of *O. maccoyi* (20 mm) is greater than those of *O. guerreroensis* n. sp. (4.4–7.3 mm) and, consequently, with more segments throughout strobila (89 vs. 31–39, respectively). In addition, neck is relatively smaller in our tapeworms (0.36–0.63) than in *O. maccoyi* (0.70–0.90) and type locally of this last species is Anguilla, Lesser Antilles, parasitizing *Anolis gingivinus* Cope (Dactyloidae) [19].

The third species of the Neotropical realm, *O. leonregagnonae*, was also described from Mexico (Oaxaca) from the intestine of *Ctenosaura pectinata* Wiegmann (Iguanidae). Nevertheless, it differs considerably from *O. guerreroensis* n. sp. in total body length (57 mm vs. 4.4–7.3 mm, respectively), as well as in number of proglottids in the strobila (98 vs. 31–39, respectively). The scolex is larger in *O. leonregagnonae* (500–800) than in *O. guerreroensis* (271–473), and both species have some differences in the reproductive systems: *O. leonregagnonae* have a greater number of testes (78–112 vs. 48–55), as well as a greater number of ovarian sublobes (31–79 vs. 3–5); finally, the vitellaria’s shape is irregular in the parasite species of *C. pectinata* [20] and ovoid in *O. guerreroensis* n. sp.

With respect to the *Oochoristica* species with mature proglottids wider than longer and distributed in the Nearctic realm, *Oochoristica islandensis* differs from *O. guerreroensis* n. sp. by total body length (15–24 mm vs. 4.4–7.3, respectively) as well as by number of proglottids along the strobila (45–69 vs. 31–39). Furthermore, *O. islandensis* has a smaller scolex (255–380 vs. 271–473, respectively) and its vitellarium is triangular, while in *O. guerreroensis* n. sp. is ovoid. Besides, testes of *O. islandensis* are divided into two clusters (with a total of 38–46 testes) contrasting with *O. guerreroensis* n. sp., which form a cluster with 48–55 testes. Finally, the number of ovarian sublobes is greater in *O. islandensis* (6–8 vs. 3–5), which was described in California, USA, parasitizing *Xantusia riversiana* Cope [21]. *Oochoristica bezyi* differs from the new species having smaller scolex (200–300 and 271–473, respectively) and suckers (88–150 × 125–175 vs. 201–301 × 127–227). Likewise, both species can be distinguished by distribution and arrangement of testes; *O. bezyi* has 22–32 testes distributed in two clusters while *O. guerreroensis* n. sp., have one cluster with 48–55. Additionally, ovary is smaller in *O. bezyi* (90–130) than in *O. guerreroensis* n. sp. (164–218), gland vitelline are triangular in *O. bezyi* and ovoid in new species and, finally, geographic distribution and type host are different in both species, since the species of Goldberg and Bursey [21] was described in California, USA as parasite of *Xantusia vigilis vigilis* Baird (Xantusiidae).

Finally, the validity of our species can be confirmed by the host distribution, due to *S. ochoterenae* is a lizard species endemic to the central region of Mexico [5, 6].

### Oochoristica Scelopori Voge & Fox, 1950

Mainly based in morphological characters, distribution and hosts identity, this material was assigned to *O. scelopori* according to [22]. Currently, *O. scelopori* has been registered in seven species of Phrynosomatidae and four of Teiidae within of 11 Mexican States [23, 24] (Figs. 1 and 2).

### Phylogeny

**Fig. 3:**
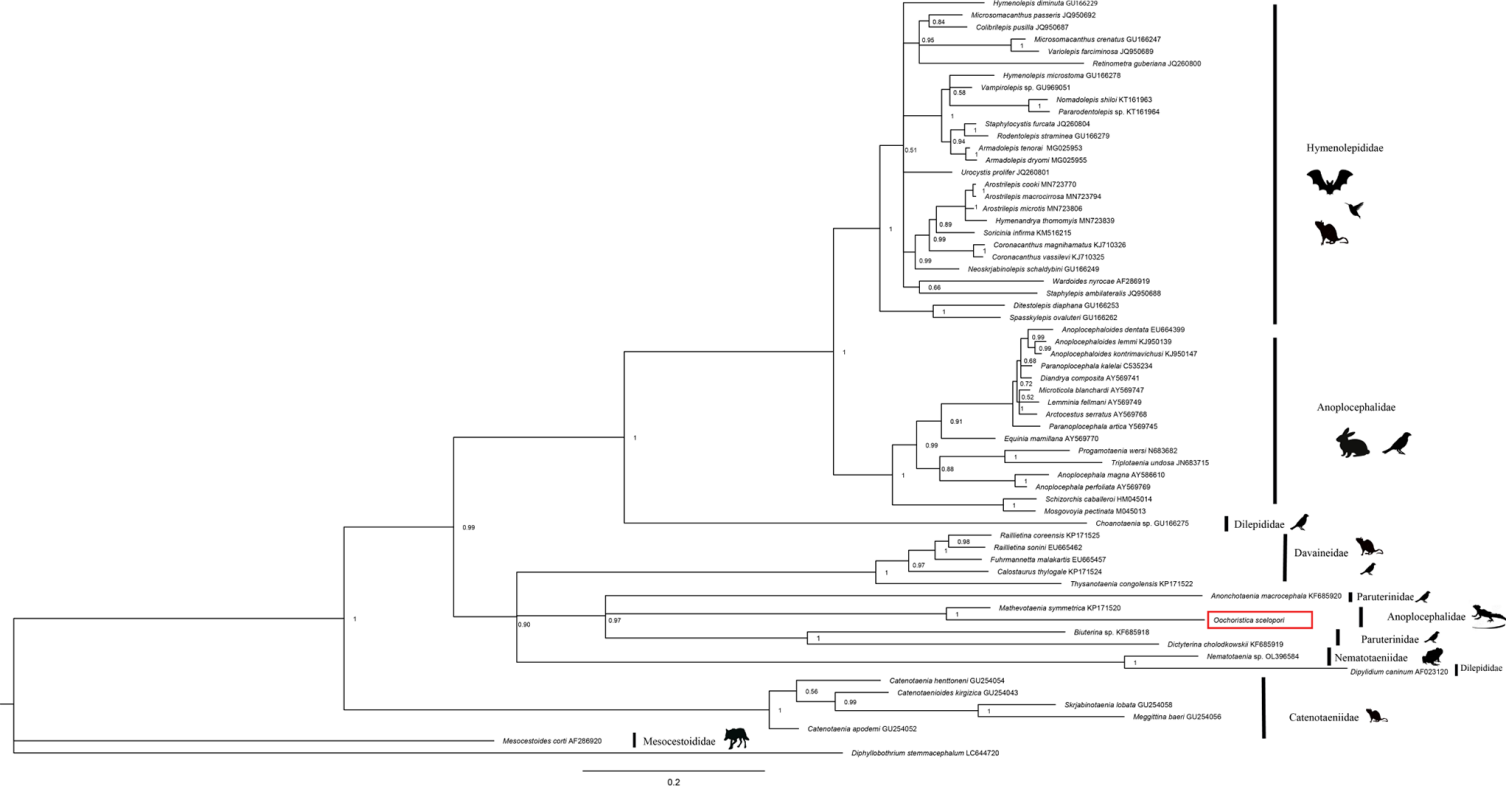
Bayesian inference phylogenetic tree of the *28 S rDNA* nuclear gene sequences of Cyclophyllidea, including the sequence belonging to *O*. *scelopori* parasite of *S. grammicus* (Tlaxcala, Mexico). Some families don’t form monophyletic groups according to the observed topology. Posterior probability is indicated at each node. GenBank access numbers are provided next to each tip name in the tree

A phylogenetic tree of Cyclophyllidea constructed in this study was based on the 28*S rDNA* nuclear gene sequences of *O. scelopori* and 63 species assigned to this order in GenBank, the inferred hypothesis form some polyphyletic groups such as Parauterinidae, Dilepididae and Anoplocephalidae. Anoplocephalidae is recovered as a non-monophyletic group formed by two clades highly supported. In particular, *Oochoristica* was recovered closely related with *Mathevotaenia*, forming a monophyletic clade; this is consistent with the old proposal to separate both genera based on segmental overlap, acraspedota in *Oochoristica* and craspedota in *Mathevotaenia* (Fig. 3).

## Discussion

Within Linstowiinae, *Oochoristica* is the most speciose genus [1, 3]. This genus presents some problems for the identification of its species, mainly due to the ambiguity of some morphological characters used, phenotypic plasticity and lack of molecular information. For example, although *O. guerreroensis* n. sp. can be morphologically differentiated from the other species of the genus based on the shape of the mature proglottids, this character is shared with 13 species worldwide, five of them in the American continent [2, 17]. A tool that would be very useful in differentiating *Oochoristica* species is molecular sequencing; however, to date, genetic material is only available in GenBank for one species parasitising the gecko *Hemidactylus brooki* Gray: *Oochoristica hemidactyli* Johri, 1955 from India [25]. Verma et al. [25] included *O. hemidactyli* in a phylogenetic context with the nuclear genes *18 S rRNA* and ITS1-5.8-ITS2 within Cyclophyllidea. The phylogeny obtained by [25] agrees with those of Mariaux et al. [1], considering that Anoplocephalidae has a polyphyletic origin, since the members that parasitize reptiles (Linstowiinae, raised to family level by [25]) nested together and those that parasitize mammals and birds (Anoplocephalinae) constituted a separate group. Our study reaches similar conclusions with the inclusion of one sequence of the *28 S* gen of *O. scelopori*. In our Bayesian Inference tree, *O. scelopori* forms a monophyletic clade (Linstowiinae) with *Mathevotaenia*, the only genus within the subfamily within molecular data. This point is particularly important because *Mathevotaenia* and *Oochoristica* can be differentiated from a morphological perspective only by the arrangement of craspedote (*Mathevotaenia*) or acraspedote (*Oochoristica*) segments in the strobili [26]. According to Bursey et al. [27] maintaining the separation of both genera based on this trait led to assigning a lizard parasite to the genus *Oochoristica* (*O. eremophilia* Beveridge, 1977) and a mammalian parasite to *Mathevotaenia* (*M. panamensis* Bursey, Goldberg & Telford, 2010). Molecular data obtained in our study seem to confirm the independence *Oochoristica* and *Mathevotaenia*. However, a more exhaustive study, including more sequences from both genera as well as a multilocus approach, will allow us to establish their taxonomic status with precision. In the present study, we add a new sequence of the 28* S rRNA* gene of *O. scelopori*, a species with wide distribution along Mexico (Sonora, Chihuahua, Durango, Guanajuato, Hidalgo, Queretaro, Zacatecas, Coahuila, CDMX, Oaxaca, and Puebla) in several species of phrynosomatid lizards [24, 28]. In this study, we characterize molecular characters from one specimen of this species found in Tlaxcala State, parasitizing *S. grammicus*.

## Data Availability

No datasets were generated or analysed during the current study.

## Declarations

### Manuscript Submission

Manuscript submitted to this journal was neither send to another journal nor any part of it has been published or is being considered for publication elsewhere in any form.
